# Archetype models upscale understanding of natural pest control response to land‐use change

**DOI:** 10.1002/eap.2696

**Published:** 2022-09-18

**Authors:** Nikolaos Alexandridis, Glenn Marion, Rebecca Chaplin‐Kramer, Matteo Dainese, Johan Ekroos, Heather Grab, Mattias Jonsson, Daniel S. Karp, Carsten Meyer, Megan E. O'Rourke, Mikael Pontarp, Katja Poveda, Ralf Seppelt, Henrik G. Smith, Richard J. Walters, Yann Clough, Emily A. Martin

**Affiliations:** ^1^ Lund University, Centre for Environmental and Climate Science (CEC) Lund Sweden; ^2^ Biomathematics and Statistics Scotland Edinburgh UK; ^3^ Stanford University, Woods Institute for the Environment, Natural Capital Project Stanford California USA; ^4^ University of Minnesota, Institute on the Environment St. Paul Minnesota USA; ^5^ Eurac Research Institute for Alpine Environment Bozen/Bolzano Italy; ^6^ Present address: Department of Agricultural Sciences University of Helsinki Helsinki Finland; ^7^ Department of Entomology Cornell University Ithaca New York USA; ^8^ Department of Ecology Swedish University of Agricultural Sciences Uppsala Sweden; ^9^ Department of Wildlife, Fish, and Conservation Biology University of California – Davis Davis California USA; ^10^ German Centre for Integrative Biodiversity Research (iDiv) Halle‐Jena‐Leipzig Leipzig Germany; ^11^ Faculty of Biosciences, Pharmacy and Psychology University of Leipzig Leipzig Germany; ^12^ Martin Luther University Halle‐Wittenberg, Institute of Geoscience & Geography Halle (Saale) Germany; ^13^ Department of Horticulture Virginia Polytechnic Institute and State University Blacksburg Virginia USA; ^14^ Department of Biology Lund University Lund Sweden; ^15^ Department of Computational Landscape Ecology Helmholtz Centre for Environmental Research – UFZ Leipzig Germany; ^16^ Leibniz University Hannover, Institute of Geobotany, Zoological Biodiversity Hannover Germany

**Keywords:** archetype, conservation biological control, crop, ecological model, landscape, land use, natural enemy, natural pest control, pest, upscale

## Abstract

Control of crop pests by shifting host plant availability and natural enemy activity at landscape scales has great potential to enhance the sustainability of agriculture. However, mainstreaming natural pest control requires improved understanding of how its benefits can be realized across a variety of agroecological contexts. Empirical studies suggest significant but highly variable responses of natural pest control to land‐use change. Current ecological models are either too specific to provide insight across agroecosystems or too generic to guide management with actionable predictions. We suggest obtaining the full benefit of available empirical, theoretical, and methodological knowledge by combining trait‐mediated understanding from correlative studies with the explicit representation of causal relationships achieved by mechanistic modeling. To link these frameworks, we adapt the concept of archetypes, or context‐specific generalizations, from sustainability science. Similar responses of natural pest control to land‐use gradients across cases that share key attributes, such as functional traits of focal organisms, indicate general processes that drive system behavior in a context‐sensitive manner. Based on such observations of natural pest control, a systematic definition of archetypes can provide the basis for mechanistic models of intermediate generality that cover all major agroecosystems worldwide. Example applications demonstrate the potential for upscaling understanding and improving predictions of natural pest control, based on knowledge transfer and scientific synthesis. A broader application of this mechanistic archetype approach promises to enhance ecology's contribution to natural resource management across diverse regions and social‐ecological contexts.

## INTRODUCTION

Worldwide, sustainable agriculture relies on integrated pest management principles to reduce crop losses to pests through a combination of ecological understanding and technological advances (Oerke, 2006). Less sustainable agricultural practices, such as extensive pesticide use (Lechenet et al., 2017), can be complemented or even replaced by natural control of arthropod pests (Holland et al., 2017; Khan et al., 2014; Tschumi et al., 2015). Natural pest control in a crop field depends on the activity of natural enemies (e.g., predators and parasitoids) and the availability of host plants for the pests (Pedigo & Rice, 2014). Both factors are controlled not only by crop management in the field but also by land‐use patterns in the landscape surrounding the crop field (Landis et al., 2000; Tscharntke et al., 2005). In theory, landscapes can thus be designed to enhance natural pest control (Bianchi et al., 2006; Chaplin‐Kramer et al., 2011). In practice, however, pest suppression and crop yields show inconsistent responses to changes in landscape composition and configuration across cases (Karp et al., 2018), with effects often being modified by diverse life histories, ecological settings, and management regimes (Dominik et al., 2018; Tscharntke et al., 2016).

Mechanistic models, that is, models of explicit causative agents, can provide understanding and predictions of ecological responses to environmental change when such responses are difficult to anticipate based on observed correlation alone (Gotelli et al., 2009; Seppelt et al., 2013). The application of classical biological control has benefited from predictions of mechanistic ecological models (Palladino, 2013). In contrast, the complexity and context sensitivity of natural pest control (Tscharntke et al., 2016) exacerbate fundamental modeling trade‐offs (Levins, 1966). Modelers have had to either sacrifice generality by realistically representing narrowly defined systems or sacrifice realism through general models of largely theoretical systems (Alexandridis et al., 2021). Consequently, existing models do not provide realistic predictions of natural pest control at landscape to global scales, where multiple crop–pest–enemy systems are involved (Seppelt et al., 2020).

Sustainability science faces similar challenges of systems complexity, heterogeneity, and context dependence (Cox, 2014; Magliocca et al., 2018; Verburg et al., 2015). Researchers are increasingly addressing these challenges using archetype analysis to identify recurrent patterns among causal relationships that shape sustainability across cases (Eisenack et al., 2019). Recurrent patterns are then translated into archetypes, that is, “context‐sensitive, generalized models of sustainability problems, dynamics or strategies with case‐level empirical validity” (Oberlack et al., 2019). For instance, Sietz et al. (2017) used similarities in social‐ecological constraints to food security in the drylands of sub‐Saharan Africa to cluster a diverse set of farming systems into groups, or archetypes, of vulnerability. They thus found that most of the studied area benefits from relatively good governance but suffers from both high remoteness and extremely dry and resource‐constrained conditions. In systems with better agricultural potential, food security is mostly threatened by high erosion sensitivity and relatively severe undernourishment. Shared determinants of vulnerability to food insecurity allow for targeted, evidence‐based promotion of strategies, such as sustainable agricultural intensification and the transfer of knowledge among farming systems grouped into the same archetype. Recurrent patterns in causal relationships can be identified in complex spatial (e.g., Messerli et al., 2016) and temporal phenomena (e.g., Levers et al., 2018). We suggest the use of an archetype approach to identify similar patterns in natural pest control, underpinning model development from landscape to global scales.

Using archetypes as context‐specific representations of general mechanisms behind natural pest control can capture essential features of agroecosystem functioning, such as feedbacks between pesticide use and pest suppression (Normile, 2013) or the nonlinear effects of landscape complexity on biodiversity (Concepción et al., 2012). Defining a reasonable number of archetypes can strike the missing balance between model generality and realism (Alexandridis et al., 2021) and contribute to the resolution of inconsistencies in natural pest control responses to agricultural management (Karp et al., 2018). Wider adoption of a mechanistic archetype approach can reduce the reliance of sustainability science on event‐oriented phenomenological models (Meyfroidt, 2016) of questionable causality (Oberlack et al., 2019) that ignore feedbacks and are, thus, associated with high policy resistance (Sterman, 2010). Moreover, predictions derived from underlying mechanisms should be more robust to changing environmental conditions than purely correlation‐based predictions (Cuddington et al., 2013).

In the following three sections, we first describe the general properties of an archetype modeling approach to the representation of geographically distant natural pest control systems that share key characteristics. Second, we demonstrate a proof of concept for such an approach by initially deriving two crop–pest–enemy archetypes from general ecological theory and available knowledge of American and African agroecosystems and then testing the ability of the two archetypes to reproduce observed responses of natural pest control to changes in landscape composition and configuration across Europe. Finally, we draw conclusions to leverage the approach for the purpose of upscaling understanding and improving prediction in agroecosystems worldwide.

## STRATEGY FOR DEFINING AND MODELING ARCHETYPES OF NATURAL PEST CONTROL

### From traits to archetypes

The definition of archetypes for heterogeneous real‐world systems requires that multiple cases of the studied phenomenon show similar responses to change and that these cases share key attributes (Oberlack et al., 2019). Natural pest control may appear to respond idiosyncratically to land‐use gradients, but the life‐history traits of pests or their enemies could mediate their responses to landscape characteristics in a predictable way (Segoli & Rosenheim, 2012). For instance, organisms in different systems often show similar responses to agricultural land‐use change, when grouped according to their dietary, dispersal, and overwintering traits (Martin et al., 2019). Similar to life‐history traits, agronomic characteristics, such as spatial and temporal in‐field crop diversity, are also expected to mediate natural pest control responses consistently across systems owing to converging crops and management practices worldwide (Malek & Verburg, 2020; Woodward & Bohan, 2013). The archetype approach can ultimately incorporate most agroecosystem properties linked to natural pest control, including climate, biogeography, and baseline levels of landscape characteristics subject to land‐use change.

Trait‐mediated similarities in natural pest control responses to land‐use gradients indicate the potential to group diverse agroecosystem components into archetypes that represent important processes behind observed patterns. Ecological theory enables such a mechanistic aggregation by linking mediating traits to underlying processes (Lavorel & Garnier, 2002; Lavorel & Grigulis, 2012; Pontarp et al., 2019). Specific values of functional (e.g., dietary, dispersal or overwintering) traits can thus describe context‐specific roles of pests and natural enemies in general processes, such as reproduction, mortality, dispersal, predation, herbivory, and environmental filtering driven by agricultural land use (Alexandridis et al., 2021). Trait‐based conceptual models of each archetype would represent the most salient roles of pests and natural enemies within these processes in the form of system components and their relationships. The level at which systems are aggregated into archetypes should allow each set of components and relationships to represent multiple systems and, at the same time, generate predictions that agree with system‐specific observations. Inclusion of agroecosystem properties other than biological traits in archetype definition will potentially improve a model's pertinence and predictive ability.

### A robust modeling framework

Reaching archetypes' full potential for improved understanding and prediction requires the use of rigorous techniques to translate trait‐based archetype components and their relationships into model variables and their interactions (Ings et al., 2009; Zakharova et al., 2019). The resulting mechanistic models can leverage established knowledge from ecological theory to produce outputs of interest (e.g., pest population levels or crop yields) from existing or anticipated system inputs (e.g., changing landscape proportion of noncrop habitat or crop rotations). The predictions of archetype models can be compared with observed responses of natural pest control to land‐use gradients across cases that correspond to each archetype. Agreement between predictions and observations would verify the archetypes' ecological basis (Overmars et al., 2007). Further model analysis can improve our understanding of the respective agroecosystems and indicate priority areas for future research (Pontarp et al., 2019).

A major challenge for model development is the typically high level of uncertainty associated with system structure, technical formulation, and model parameterization. All of these sources of uncertainty can be constrained by limiting the complexity of ecological models (Cuddington et al., 2013), with the added benefit of enhancing model transferability between systems (Yates et al., 2018). The first source of uncertainty, related to system structure, can be addressed by using allometric relationships to predict trophic interactions (Curtsdotter et al., 2019). The well‐established use of body size can be complemented with other biological traits (Wood et al., 2015; Woodward & Bohan, 2013) to compensate for losses in predictive power as trophic complexity increases (Curtsdotter et al., 2019; Jonsson et al., 2018). The second source of uncertainty, regarding model formulation, can be tackled through ensemble forecasting by multiple models (Araújo & New, 2007). Alternatively, one may only consider “robust theorems” (Levins, 1966), that is, shared predictions of models developed independently but conditioned on the assumptions that define each archetype. We adopt the latter approach in selected examples (see below) because it offers more flexibility with respect to employed techniques, encompassing qualitative and quantitative models. Qualitative mathematical modeling requires no parameter estimation, thereby circumventing the third source of uncertainty, associated with parameterization, which is prominent in quantitative modeling (Levins, 1998). Therefore, we independently develop qualitative and quantitative models for example archetypes but acknowledge that these represent just two of the many possible models for each.

### Applying the archetype approach

The development of mechanistic models for each archetype, as illustrated in the following section, is an iterative process that can be divided into four main stages (Sterman, 2010). (1) The process starts with the articulation of the problems that need to be addressed by an application of the archetype approach. Such problems typically arise from an inability of existing models to explain observations. Problem description indicates the spatiotemporal scales of the focal system and system components. (2) Then hypotheses are combined into a mechanistic archetype, with the goal of explaining observations across systems. Here, previously identified system components are linked through causal relationships based on available mechanistic understanding. (3) The compiled hypotheses are then translated into system variables and their interactions, within models that aim to reproduce observed system patterns. The characteristics of these model elements can vary depending on the adopted modeling technique. (4) Finally, the models' predictive ability is tested against observations in order to evaluate the underlying hypotheses and improve our understanding of the system. These observations should be as independent as possible from sources of mechanistic understanding used in model development in order to test the applicability of archetype models across systems.

## APPLYING THE ARCHETYPE APPROACH—A WORKED EXAMPLE

### The problem

Individual components of natural pest control, and their responses to landscape‐scale land use, are often studied in isolation, impeding our understanding of observed patterns and reliable prediction. A recent synthesis of natural pest control observations across Europe (Martin et al., 2019) indicates significant, trait‐mediated responses of involved organisms to changes in landscape composition and configuration. On the one hand, natural enemies that are generalists in their feeding behavior and move actively by flight or on the ground between crop and noncrop habitats increase in abundance in response to increasing noncrop habitat proportion and field edge density (Table 1). This observation is consistent with our understanding of the facilitative role of landscape complexity for natural enemy dispersal and resource provision (Tscharntke et al., 2012). In contrast, dietary specialist enemies that may disperse passively by wind tend to decrease in abundance or show no response to increasing landscape complexity. Therefore, dietary and dispersal traits appear to interactively dictate the responses of natural enemies to land‐use gradients, but the underlying mechanisms are not clear.

**TABLE 1:** Observed trait‐mediated responses of pest and natural enemy abundances to changes in landscape composition and configuration across Europe, including different baseline levels of these landscape characteristics, in cases where such differences were linked to significant divergence in abundance responses (Martin et al., 2019). Pests overwinter in crop fields (resident) or noncrop habitats (transient). Natural enemies of pests disperse passively or actively; the former have narrow (specialist) and the latter broad (generalist) feeding preferences. Responses of these organisms to increasing landscape proportion of noncrop habitat and density of field edges are given as upward and downward facing arrows for increases and decreases, respectively. Lack of responses is illustrated with dashes.

Abundance responses	Increasing noncrop habitat		Increasing edge density	
Resident pest	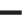		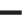	
Transient pest	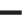		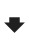	
Passively dispersing specialist enemy	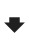 ^a^	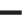 ^b^	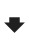 ^c^	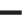 ^d^
Actively dispersing generalist enemy	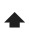		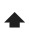	

^a^ In landscapes with relatively low density of field edges.

^b^ In landscapes with relatively high density of field edges.

^c^ In landscapes with relatively low proportion of noncrop habitat.

^d^ In landscapes with relatively high proportion of noncrop habitat.

Martin et al. (2019) further observed distinct responses of pest abundances to land‐use gradients, depending on the explanatory variable and the pests' overwintering strategy, that is, in crop fields (resident) or noncrop habitats (transient). Only a higher density of field edges in the landscape, but not a higher proportion of noncrop habitat, leads to a lower abundance of transient pests (Table 1). Resident pests, in contrast, do not respond to either land‐use variable. The need of transient pests to move between crop and noncrop habitats appears to mediate their distinct responses (Tscharntke et al., 2012). However, the lack of impact by noncrop habitat amount cannot be explained by these traits alone and may be related to other traits, such as invasive status (Tamburini et al., 2020). Elucidating differences in pest and natural enemy responses and assessing the degree to which differences are influenced by and feedback to other system components, including crop yields, requires a system perspective that encompasses multiple trophic levels.

### Combining hypotheses into archetypes

Among the many properties of complex agroecosystems that can shape natural pest control around the world, we here focus on a few key life‐history traits. In the following examples, we present two archetypes that incorporate divergent natural enemy dietary and dispersal behaviors and that differ in pest overwintering strategies, along with system elements related to each strategy. Besides facilitating the following illustration of archetype definition and model development, this choice also assesses the ability of the archetype approach to parsimoniously reproduce markedly different pest and natural enemy responses to change, through simple, minimally different models.

**FIGURE 1:**
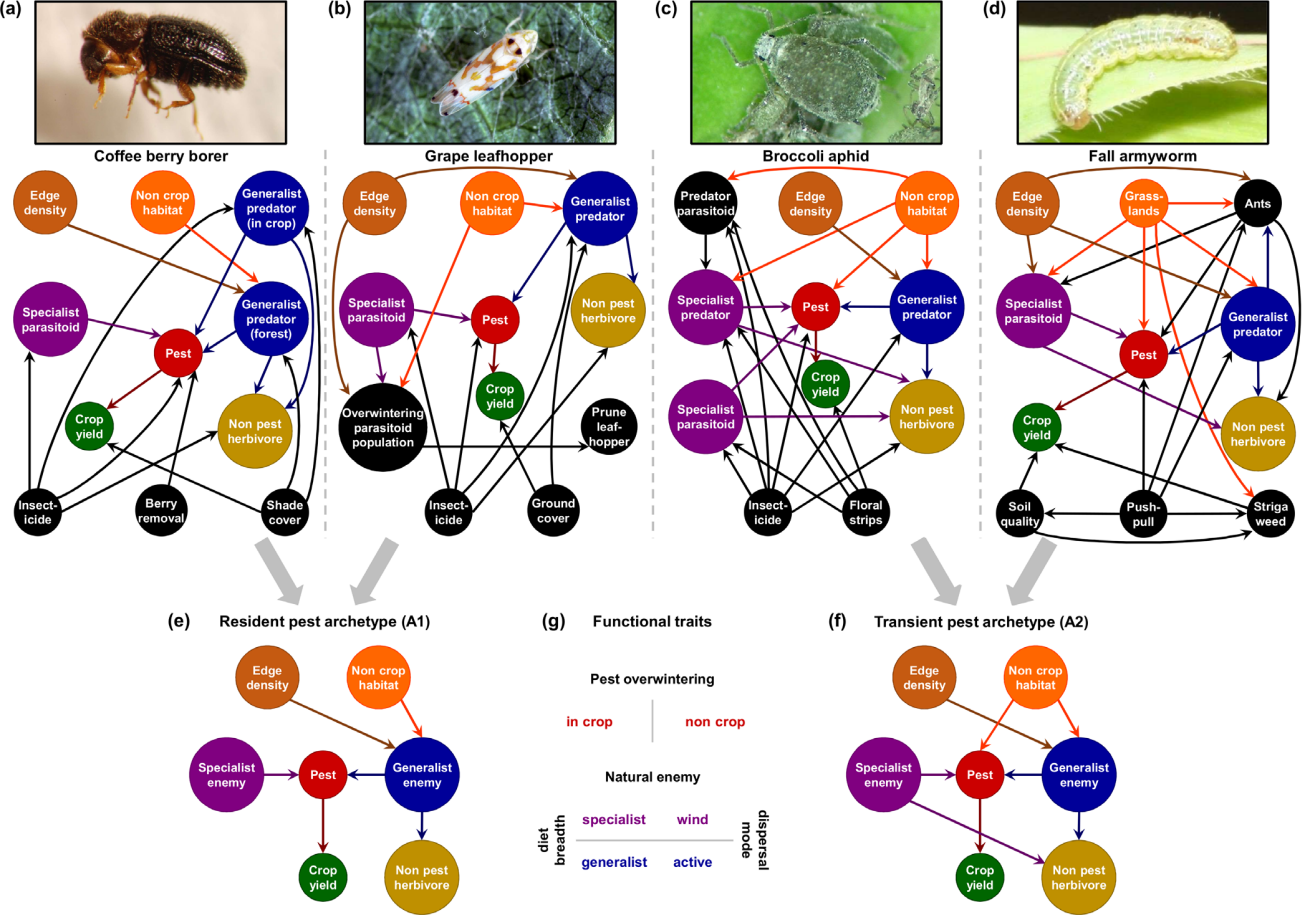
Conceptual models based on expert knowledge of systems of natural control of (a) coffee berry borer (*Hypothenemus hampei*) in Latin America, (b) grape leafhopper (*Erythroneura* spp.), (c) broccoli aphid (*Brevicoryne brassicae*) in North America, and (d) fall armyworm (*Spodoptera frugiperda*) in sub‐Saharan Africa. Arrows indicate diverse causal relationships, pointing from cause to effect, and colors identify system components with pertinent similarities. Conceptual models of Archetypes A1 and A2 (e and f) aim at representing the defining components of multiple systems with respect to natural pest control and crop yield responses to changes in landscape characteristics (noncrop habitat proportion and field edge density). (g) Model simplification is based on theoretical expectations regarding the role of pest overwintering and enemy dietary and dispersal traits in natural pest control. See main text for details. Photo credits: (a) Daniel S. Karp, (b) Jack Kelly Clark, courtesy University of California Statewide IPM Program, (c) Andrew Jensen, and (d) Yann Clough.

Mechanistic understanding of natural pest control in several American and African agroecosystems was elicited from experts through participatory modeling techniques (Fulton et al., 2015) toward the formulation of four conceptual models (Figure 1a–d). These detailed models were then simplified into two archetypes of crop–pest–enemy interactions (Figure 1e,f). Simplification was achieved by only retaining system components whose traits appeared to mediate natural pest control responses to land‐use gradients in Europe (Martin et al., 2019) (Figure 1g). We then modeled theory‐anticipated functional roles of organisms with specific values of these traits. We focused on these traits and system components in order to capture important mechanisms that shape natural pest control across systems and to allow comparison of archetype model predictions with observations of pests and natural enemies with the respective trait values from geographically distant systems (Table 1). These objectives are further facilitated by using trait values that are well represented in empirical observations of natural pest control, while still defining archetypes that are general enough to allow the use of ecological theory and available expert knowledge.

The two conceptual models (Figure 1e,f) represent resident pest (termed A1) and transient pest (termed A2) archetypes. A resident pest (A1) stays in crop fields throughout the year, whereas a transient pest (A2) moves to noncrop habitats when crop resources are unavailable or noncrop host plants are required (indicated as “overwintering,” even for systems that lack winter per se). Pest populations grow by feeding on a specific crop without density dependence, that is, reflecting the relative abundance of the crop. In‐field agricultural management practices, such as intercropping or cover cropping, are not considered. Both archetypes include natural enemies that specialize on the pest's taxonomic family and disperse with the help of the wind (specialist enemy), as well as other natural enemies that feed on a variety of pest and nonpest herbivores and disperse by active movement (generalist enemy).

The specialist enemy in Archetype A1 is exposed to abundant pest prey in the crop, so its density reflects pest relative abundance. In contrast, pest migration to noncrop habitats in Archetype A2 forces the specialist enemy to either seek other prey (usually of the same family) within the crop or move to more species‐rich noncrop habitats. This hypothesis is supported by the observed behavior and host range of the main braconid parasitoids of pests in the Archetype A2 example agroecosystems: the broccoli aphid in North America (Pike et al., 1999) and the fall armyworm in sub‐Saharan Africa (Agboyi et al., 2020). As a result, the specialist enemy in Archetype A2 diversifies its diet with nonpest herbivores, thereby reducing its dependence on the pest. We note that this group is nevertheless “specialized” compared to generalists that are able to prey on a wide range of taxonomic families. Higher proportions of noncrop habitat in the landscape enhance organisms that rely directly on noncrop resources, that is, generalist enemies in both archetypes and the pest in Archetype A2. Field edges also provide such resources, along with interfaces for spillover between and into crops, particularly to generalist enemies actively dispersing in short distances (Tscharntke et al., 2012).

### Qualitative and quantitative models

**FIGURE 2:**
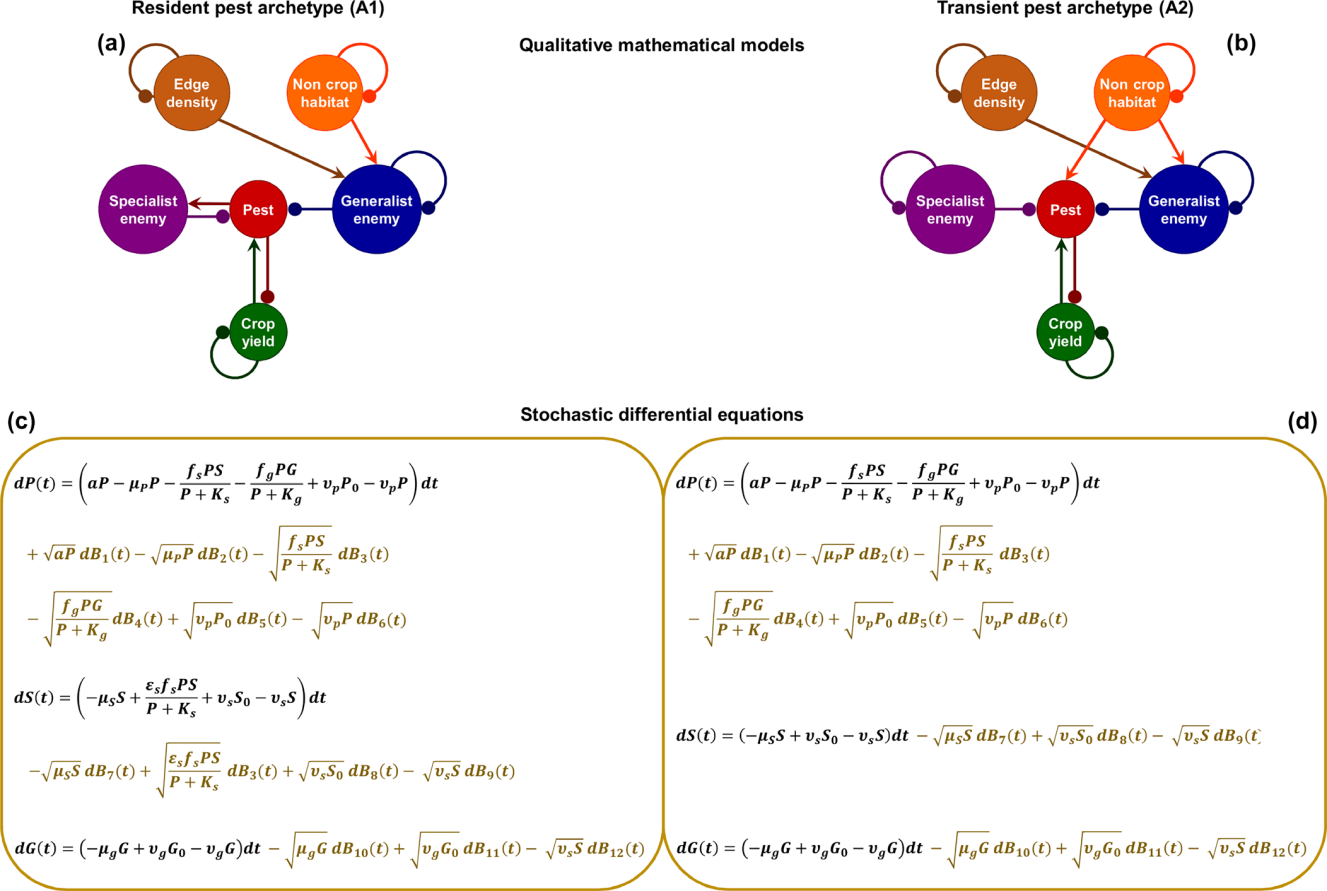
Qualitative and quantitative modeling of Archetypes A1 (left‐hand panels) and A2 (right‐hand panels) based on respective conceptual models (Figure 1e,f). Note that both modeling approaches represent a subset of the conceptual models' variables considered as necessary. Qualitative mathematical models (a and b) are shown as signed digraphs, that is, networks of directed interactions, including loops of self‐effects, with arrows for positive and dots for negative signs. Stochastic differential equations (c and d) represent the dynamics of pest (P), specialist (S), and generalist (G) enemy populations. For details, see the main text. Stochastic terms are shown in gold font, where ${\mathrm{dB}}_{i}\left(t\right)\;$ represent Gaussian white noise and, for each t and i=1,…12, are independently drawn from $N\left(0,\sqrt{\mathrm{dt}}\right)$.

We developed mechanistic models of Archetypes A1 and A2, with the goal of yielding testable predictions from each set of hypotheses. We independently formulated qualitative (Figure 2a,b) and quantitative (Figure 2c,d) models based on the same basic assumptions for each archetype but using system simplifications that are specific to the two modeling techniques. Qualitative mathematical models (Levins, 1998) represent interactions among crop yield and populations of pests and natural enemies, as well as the proportion of noncrop habitat and field edge density in the landscape (Figure 2a,b). This technique has been applied to improve understanding and prediction of classical biological control of crop pests (e.g., Levins, 1969; Levins & Schultz, 1996). Signed digraphs (networks depicting the direction and sign of interactions among variables) represent the structure of a system as a whole. The matrix representation of a signed digraph is a qualitatively specified (i.e., consisting of −1, 0, and 1) community matrix (linearization of a Lotka–Volterra equation at an equilibrium point) (Puccia & Levins, 1985). Standard analysis of this qualitative matrix (Dambacher et al., 2002) predicts the equilibrium responses of system variables to sustained increases or decreases in landscape proportion of noncrop habitat and field edge density (Appendix S1).

We implemented quantitative models as sets of stochastic differential equations (e.g., Walton et al., 2016). For simplicity, in what follows we describe the deterministic part of the model for Archetype A1 in order to illustrate its basic structure (for the full stochastic A1 and A2 equations, which add structured variability to the deterministic models, see Figure 2c and Appendix S1):

(1) $$\frac{\mathrm{dP}}{\mathrm{dt}}=\mathrm{aP}-\mu_{P}P-\frac{f_{s}\mathrm{PS}}{P+K_{s}}-\frac{f_{g}\mathrm{PG}}{P+K_{g}}+\upsilon_{p}P_{0}-\upsilon_{p}P,$$

(2) $$\frac{\mathrm{dS}}{\mathrm{dt}}=-\mu_{S}S+\frac{\varepsilon_{s}f_{s}\mathrm{PS}}{P+K_{s}}+\upsilon_{s}S_{0}-\upsilon_{s}S,$$

(3) $$\frac{\mathrm{dG}}{\mathrm{dt}}=-\mu_{g}G+\upsilon_{g}G_{0}-\upsilon_{g}G,$$

where P, S, and G are the numbers of pests, specialist natural enemies, and generalist natural enemies in the crop, which suffer from natural mortality at rates $\mu_{P}$, $\mu_{S}$, and $\mu_{G}$, respectively. Pests have a population growth rate of a and suffer predation or parasitism at a rate of $\left(f_{s}\mathrm{PS}\right)/\left(P+K_{s}\right)$, representing a functional type II response, which describes the ability of consumers to detect and consume pests (Holling, 1959). For the parameters shown, the average time taken to handle a prey item is $1/f_{s}$, and the rate at which specialist enemies encounter prey per unit density is $f_{s}/K_{s}$. Specialist enemies convert consumed pests with efficiency $\varepsilon_{s}$. Similarly, generalist enemies prey on the pests at rate $(f_{g}\mathrm{PG})/\left(P+K_{g}\right)$, where $f_{g}$ and $K_{g}$ describe the generalist enemies' ability to detect and consume the pests. Note that, owing to generalist enemies' direct reliance on noncrop resources, we assume that the population size of this natural enemy group is not increased by the consumption of pests. Although likely not often realistic, this assumption allows us to represent the distinction between generalist and specialist natural enemies with a parsimony that suits our demonstrative purposes. More generally, the influence of noncrop resources is described in terms of both inflow and outflow of organisms, for example, for pests $\upsilon_{p}P_{0}$ and $\upsilon_{p}P$, respectively. The parameters $P_{0}$, $S_{0}$, and $G_{0}$ represent the amounts of pests, specialist enemies, and generalist enemies, respectively, that noncrop habitats can sustain. The parameters $\upsilon_{p}$, $\upsilon_{s}$, and $\upsilon_{g}$ describe the connectivity between crop and noncrop habitats, which is experienced differently by pests, specialist enemies, and generalist enemies, respectively. The model for Archetype A2 is the same as for A1, except that we assume that the reproduction of specialist enemies does not depend on pests because pests that overwinter in noncrop habitat expose natural enemies to a more diverse diet. This is equivalent to assuming $\varepsilon_{s}=0$ (Figure 2d).

Changes in the landscape proportion of noncrop habitat and field edge density are imposed on the A1 quantitative model by changing $G_{0}$ in the former case and both $G_{0}$ and $\upsilon_{g}$ in the latter. This is the same in Archetype A2, except that changes in the proportion of noncrop habitat are imposed by also changing $P_{0}$. Changes in $G_{0}$ and $P_{0}$ represent the impacts of varying the quality or quantity of noncrop habitat on generalist natural enemies and pests. Quality could be enhanced, for example, by habitat improvement, but changing the quantity of noncrop habitat might reduce crop area. Our models represent pest and natural enemy density within the crop and, therefore, do not explicitly account for a trade‐off between the amount of noncrop habitat and crop area. The values of the model parameters were varied within realistic ranges based on expert knowledge of representative systems worldwide (Appendix S1).

### Model testing—Shared qualitative/quantitative predictions

Identical main hypotheses behind each archetype's pair of models resulted in consistent predictions for both qualitative and quantitative models, except for responses of the resident pest in Archetype A1 to changes in landscape characteristics (Table 2). This discrepancy stems from the quantitative model's explicit representation of crop inflow and outflow of organisms, which carries more information but requires additional hypotheses on arthropod dispersal. Still, the difference is marginal because the qualitative model predicts no change, compared to the quantitative predictions of a slight decrease (Appendix S1). In what follows, we assess the models' underlying hypotheses and their transferability across systems by comparing model predictions in response to varied parameter values with observations of natural pest control systems in Europe along land‐use gradients (Martin et al., 2019).

**TABLE 2:** Shared qualitative and quantitative predictions from models of Archetypes A1 and A2 in response to parameter variation within globally realistic value ranges. Equilibrium responses of pest, specialist and generalist enemy abundances to imposed increases in landscape proportion of noncrop habitat and density of field edges are given as upward and downward facing arrows for increases and decreases, respectively. Predictions of no response are illustrated with horizontal lines. Tildes indicate diverging response predictions (here, qualitative models predict no change, while quantitative models predict slightly decreasing abundances).

Abundance responses	Increasing noncrop habitat	Increasing edge density
Resident pest archetype (A1)		
Pest	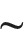	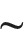
Specialist enemy	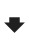	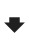
Generalist enemy	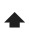	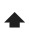
Transient pest archetype (A2)		
Pest	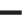	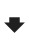
Specialist enemy	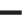	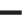
Generalist enemy	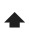	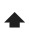

Shared qualitative and quantitative predictions for both the resident (A1) and transient (A2) pest archetypes (Table 2) agree with observations of increasing generalist enemy abundances in response to an increasing proportion of noncrop habitat and field edge density in the landscape (Table 1). Archetype A1 models further show the specialist enemy being outcompeted by the generalist and, hence, reducing in numbers. In contrast to A1, access to diverse resources in A2 allows the specialist enemy to maintain its abundance. Therefore, natural enemies that are considered broadly as specialists but exhibit varying degrees of specialization in response to different pest overwintering strategies show contrasting responses to the same changes in landscape characteristics. These predictions may explain the weak signal from impacts of land‐use gradients on the combined, but of varying specialization degree, group of specialist enemies across Europe found by Martin et al. (2019).

A1 predictions of lower specialist enemy abundance with increasing landscape complexity lead to less effective control of the pest, despite increasing generalist enemy abundance (Table 2). The predicted lack of substantial pest responses agrees with observations of no significant change in resident pest abundances in response to varying landscape characteristics (Table 1). A2 models predict no impact of noncrop habitat changes on the pest despite its use of noncrop resources, owing to conflicting influences directly on the pest (positive) and through the generalist enemy (negative). In contrast, increasing field edge density enhances the generalist enemy but not the pest, leading to effective pest control and lower transient pest abundance. The predicted distinct responses of transient pests to changes in landscape composition and configuration are a prominent pattern in observations across Europe (Martin et al., 2019). Consequently, reducing field sizes in landscapes dominated by the transient pest archetype, such as Swedish cropland with spring‐sown cereals attacked by aphids (Östman et al., 2003), is a land‐use strategy predicted to sustain crop yields independently of other management practices (Appendix S1).

To summarize, our example application illustrates the use of crop–pest–enemy archetypes for the context‐sensitive representation of general ecological mechanisms. Different assumptions regarding pests' overwintering strategy result in striking differences in predicted responses to land‐use gradients, in agreement with observations that transcend geography. Potential benefits extend beyond elucidating inconsistencies in observed patterns of natural pest control, toward generating robust predictions about quantities, such as avoided pest damage or increased crop yield, that are difficult to measure accurately across agroecosystems (Holland et al., 2017).

## LEVERAGING ARCHETYPES TO MODEL NATURAL PEST CONTROL AT LANDSCAPE TO GLOBAL SCALES

### Exploiting the potential of archetypes

The preceding examples show how crop–pest–enemy archetypes can mobilize a broad range of available knowledge to explain apparently contradictory responses of natural pest control to the same management intervention. We transfer expert knowledge across agroecosystems and synthesize general ecological theory to build archetypes that occupy an intermediate level of generality between these two extremes (Meyfroidt et al., 2018). The resulting mechanistic understanding applies to several systems, but it is context‐specific and, hence, more nuanced and with a greater potential for consistency across cases than general theories (Levins, 2005). For example, we show how exploitative competition among natural enemies can be rendered context‐sensitive by divergent overwintering strategies of pests.

The context of application can be extended beyond the functional traits of focal organisms to include variables such as baseline landscape characteristics or agricultural management regimes. For instance, simpler landscapes can be assumed to favor organisms represented by the resident pest archetype. More complex landscapes could similarly be associated with the transient pest archetype. Overrepresentation of each set of organisms in the respective landscape context would justify targeted application of the two archetypes to systems with both specific trait values and baseline landscape characteristics. In this case, the predicted responses of specialist enemies from the A1 and A2 archetype models (Table 2) match observations from relatively simple and complex landscapes, respectively (Table 1). Therefore, defining the context of application in terms of functional traits and landscape complexity can provide a testable explanation of the variable responses of specialist enemies to land‐use gradients across Europe (Martin et al., 2019).

Mathematically rigorous, testable explanations of seemingly idiosyncratic patterns, as illustrated by the example archetypes, are required for mainstream natural pest control in agricultural management worldwide (Kleijn et al., 2019). By facilitating the application of mechanistic modeling in more cases, archetypes can explain inconsistencies in the responses of natural pest control to changing landscapes (Karp et al., 2018) and prompt empirical research that distinguishes between competing hypotheses. Because changes in land use and climate are predicted to increase crop losses to insect pests (Deutsch et al., 2018) and hamper natural pest control worldwide (Raven & Wagner, 2021), mechanistic archetype models can increase predictive robustness by not only considering direct biological impacts but also modulating biotic interactions and feedbacks with agricultural management.

Agricultural landscapes often consist of mosaics of different crops that host pests and natural enemies with different characteristics. Archetypes representing major crop–pest–enemy combinations provide the building blocks for upscaling natural pest control modeling across landscapes. Predictions of farming‐system‐wide natural pest control potential will facilitate management of this ecosystem service through the design of agricultural landscapes, as well as the inclusion of natural pest control in frameworks that bridge ecology and agroeconomics (e.g., Seppelt et al., 2020). An archetype approach to modeling natural pest control would be a valuable addition to existing tools that use detailed land‐use information to map ecosystem services across landscapes (Sharp et al., 2014) or to global assessments of nature's contributions to people under different scenarios of environmental change (Chaplin‐Kramer et al., 2019).

### Toward a global set of natural pest control archetypes

**FIGURE 3:**
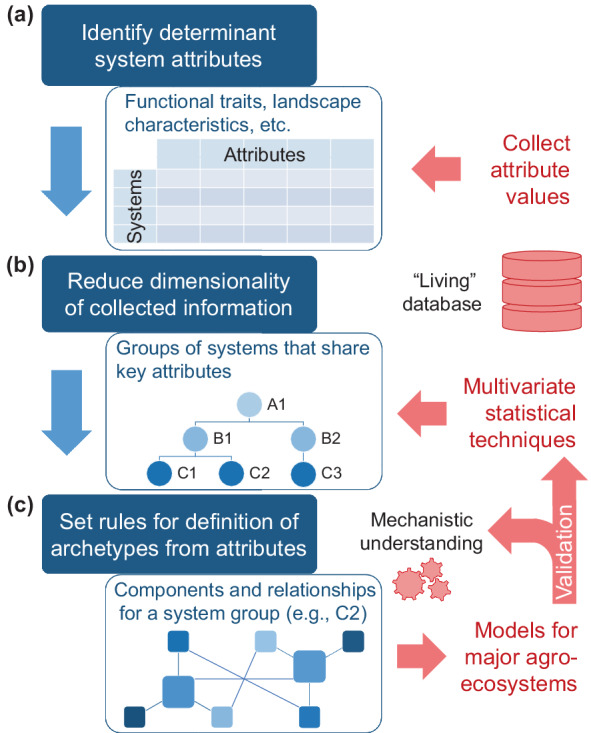
Steps toward operationalizing the definition of archetype models of natural pest control. (a) First, general attributes of natural pest control systems that determine their behavior should be identified and relevant values collected across systems. A “living” database will allow the contribution of empirical information by researchers around the world. (b) Then, multivariate statistical techniques will reduce the dimensionality of the collected information by identifying groups of systems, potentially structured hierarchically, that share key attributes. Mechanistic understanding based on ecological theory and expert knowledge will link group attributes to specific ecological processes. (c) Finally, a standardized set of rules should use these processes to define archetypes for all combinations of system attribute values. These archetypes will comprise system components and their relationships, which will be translated into elements of mechanistic models for all major agroecosystems. Validation of these models against independent observations will evaluate available mechanistic understanding and dictate the need to redefine system groups and their archetype models.

The coordination of archetype definition across agroecosystems will generate archetypes with precisely assigned contexts of application and minimize the effort required for the development of their models. We suggest a research agenda that builds on our examples to operationalize the definition of archetypes globally by taking the following steps:

1. *Identify determinant system attributes*. Example archetypes developed here are based on evidence on the mediating role of life‐history traits in natural pest control responses to land‐use gradients in Europe. Similarly, we can identify functional traits, landscape characteristics, and other attributes that play a significant role in the behavior of natural pest control worldwide and collect relevant values across agroecosystems (Figure 3a). A modeled system can thus expand from a network of interacting organisms to a more inclusive set of agroecological components. Co‐occurring crops, pests, and natural enemies from recently compiled large‐scale data sets (e.g., Dainese et al., 2019; Karp et al., 2018) provide a starting point for the identification of natural pest control systems worldwide. It should be possible to explicitly link system attributes to specific processes underlying the behavior of natural pest control, based on ecological theory and expert knowledge (Lavorel et al., 2007). Empirical studies that build consensus on drivers of behavior across systems and identify attributes with cross‐system explanatory potential (e.g., Martin et al., 2019; Tamburini et al., 2020) are a key resource for this task.
2. *Reduce dimensionality of collected information*. In the preceding examples, expert knowledge of diverse agroecosystems is rather arbitrarily simplified using trait‐based theoretical expectations. In a more systematic approach, the dimensionality of the previously collected information can be reduced using multivariate statistical techniques (Figure 3b). The selection of specific techniques will depend on the nature of collected information. For instance, the functional traits of crops, pests, and natural enemies can be used to cluster a diverse set of organisms into archetypes with distinct combinations of trait values. These values should describe the respective organisms' roles in trait‐associated processes, such as feeding, dispersal, and overwintering in the previously given examples. These processes can then inform the mechanistic representation of each archetype in terms of system components and their relationships (Boulangeat et al., 2012). Hierarchical classification of agroecosystem components linked to natural pest control (Sietz et al., 2017) has the advantage of flexible, case‐specific determination of the aggregation level in subsequent applications, depending on the model output or spatiotemporal scales of interest and knowledge availability.
3. *Set rules for defining archetypes based on attributes*. The last step should establish a standardized, dynamic set of rules for the definition of archetypes and their models based on combinations of trait values, landscape characteristics, and other system attributes (Figure 3c). These rules will allow researchers to place any real‐world system within or, if necessary, outside the previously reduced space of system attributes. In the case of systems from world regions that are underrepresented in the collected information, expert knowledge can be used to either explore close associations with existing system groups or independently develop conceptual models, similar to our example archetypes. In either case, rules should ensure the translatability of system‐specific attributes into system components and relationships, toward model variables and interactions. The four stages of model development outlined in *Applying the archetype approach* provide a potential blueprint for establishing such a rule set. Observed responses of natural pest control to land‐use gradients across systems (e.g., Dainese et al., 2019; Karp et al., 2018) can then be compared with qualitative or quantitative model predictions to assess each archetype's validity. An iterative process of model development and validation could identify the optimal set of archetypes worldwide (Alexandridis et al., 2017; Hérault, 2007).

## OUTLOOK ON ARCHETYPES

As the scope of natural pest control archetypes broadens, data establishing their empirical basis can take the form of a “living” database of crop–pest–enemy combinations and associated traits, along with environmental variables, such as landscape or climate characteristics, management regimes, and biogeographic regions. A consistent coding for this database will lower the bar for empirical researchers and practitioners willing to contribute with case‐specific knowledge. For instance, climate scientists, geographers, agronomists, entomologists, and farmers from different parts of the world will be able to feed information on rainfall, land use, crop yield, pest abundance, and pesticide application into the database in a standardized format. Data analysis will allow for the definition of archetypes at different scales, for example, regional or global. Experts will then be able to use available information to place a natural pest control system of interest among identified archetypes and apply the respective model or develop a new one. Such data will facilitate dynamic archetype definitions and systematic evaluation of the mechanistic understanding that archetypes are hypothesized to carry. Keeping this framework sufficiently flexible to constantly incorporate new knowledge will require an iterative and participatory research axis involving experts and practitioners.

The need for general modeling approaches that account for the context of application extends to many environmental areas, including major land‐use issues, such as deforestation and desertification, and their interactions with climate change (Dale, 2003). An enrichment of the archetype approach with ecological principles promises to improve the mechanistic basis of models of land‐use archetypes and broaden their predictive scope (Václavík et al., 2013). A wide adoption of an archetype approach to ecological modeling will increase the use of models with similar basic assumptions and comparable output. The ensuing potential for knowledge synthesis is particularly needed in the fragmented discipline of ecology (McGill, 2010). Furthermore, context specificity reduces the effective degrees of freedom, allowing for more robust knowledge transfer. This is crucial in the case of persistent data shortages, for example, in regions where the highest projected impacts from cumulative environmental change coincide with the least studied social and ecological systems (Beckmann et al., 2019).

## CONFLICT OF INTEREST

We have no conflicts of interest to disclose.

## Supporting information


Appendix S1
