# Supplementary material for: Archetype models upscale understanding of natural pest control response to land‐use change
Source: Ecol Appl. 2022 Sep 18;32(8):e2696. doi: 10.1002/eap.2696 (PMC10078142; doi:10.1002/eap.2696)
Supplement: Supplementary file 1 — Appendix S1 [file EAP-32-0-s001.pdf]

Ecological Applications

Archetype models upscale understanding of natural pest control response to land-use change

Nikolaos Alexandridis, Glenn Marion, Rebecca Chaplin-Kramer, Matteo Dainese, Johan Ekroos, Heather Grab, Mattias Jonsson, Daniel S. Karp, Carsten Meyer, Megan E. O'Rourke, Mikael Pontarp, Katja Poveda, Ralf Seppelt, Henrik G. Smith, Richard J. Walters, Yann Clough, Emily A. Martin

Appendix S1

S1 Qualitative mathematical models

The community matrix describes a biological community's internal dynamics. It consists of the partial derivatives of each community member's population growth rate with respect to each community member's abundance (Novak et al., 2016). This matrix can be specified qualitatively (i.e., with entries of + 1, –1 and 0), describing only the direction and sign of interactions among community members. Assuming that the modelled system is stable (i.e. able to return to its prior state following a pulse perturbation), the classical adjoint of the negative qualitatively specified community matrix describes the direction of the equilibrium abundance responses of community members to press perturbation that impacts the system through each of its variables (Dambacher et al., 2002). A system's stability is typically supported by its tendency to persist in the face of disturbance; lack of stability requires time averaging methods for qualitative predictions of perturbation responses (Puccia and Levins, 1985).

Dambacher et al. (2003) distinguish between qualitative mathematical models with unambiguous stability or instability, from conditionally stable models. They define stability criteria for the latter category, and divide its models into two classes, with the respective metrics that quantify their stability potential. Instability in class II models is characterized by overcompensation and oscillations in response to disturbance, due to feedback at higher system levels overwhelming feedback at lower levels. This case can be assessed by measuring the relative balance of feedback at different system levels. The stability of class I models is jeopardized by positive feedback dominating feedback at the highest system level, leading to community change (e.g. disappearance of a community member) if the system is disturbed. The potential for stability of class I models is determined by their weighted feedback (wF) at the highest system level (wF_n, where n is the number of variables in a system) (Dambacher et al., 2003). wF_n values that tend toward −1 indicate a high potential for the variables of a system in equilibrium to converge toward their original levels following a pulse perturbation. Values that tend toward 1 indicate a high potential for post-perturbation divergence from these levels. Values near 0 show high ambiguity with respect to the system's stability potential. Stable systems can be further analysed to predict the sign of equilibrium responses of system variables to sustained increase or decrease, i.e. a press perturbation, in each variable (Dambacher et al., 2002).

We draw signed digraphs, and from them derive qualitatively specified community matrices $\mathbf{A}^{\circ}_{1}$ and $\mathbf{A}^{\circ}_{2}$ for archetypes A1 and A2, respectively (Tables S1, S2), with the digraph editor software PowerPlay, version 2.0 (Westfahl et al., 2002). The stability of the two models is assessed with a program for the analysis of qualitatively specified community matrices (https://esapubs.org/archive/ecol/E083/022/), using the technical computing software Maple 2017.3 (Maplesoft, 2017). Both models are identified as

class I models, with *wFn* values of −1 (Table S3), indicating unambiguous stability for the respective systems. This allows prediction of the sign (if any) of equilibrium responses of system variables to sustained change in each system variable (Tables S4, S5), by calculating the classical adjoint of the negative $\mathbf{A}^{\circ}_1$ and $\mathbf{A}^{\circ}_2$ matrices (Dambacher et al., 2002).

Table S1 Qualitatively specified community matrix $\mathbf{A}^{\circ}_1$ for the A1 archetype. In a system of Lotka–Volterra equations representing the growth rate functions of the listed variables, each table value corresponds to the sign of the partial derivative of the row variable with respect to the column variable, at an equilibrium point of the system.

	Crop yield	Pest	Specialist enemy	Generalist enemy	Edge density	Non-crop habitat
Crop yield	-1	-1	0	0	0	0
Pest	1	0	-1	-1	0	0
Specialist enemy	0	1	0	0	0	0
Generalist enemy	0	0	0	-1	1	1
Edge density	0	0	0	0	-1	0
Non-crop habitat	0	0	0	0	0	-1

Table S2 Qualitatively specified community matrix $\mathbf{A}^{\circ}_2$ for the A2 archetype. In a system of Lotka–Volterra equations representing the growth rate functions of the listed variables, each table value corresponds to the sign of the partial derivative of the row variable with respect to the column variable, at an equilibrium point of the system.

	Crop yield	Pest	Specialist enemy	Generalist enemy	Edge density	Non-crop habitat
Crop yield	-1	-1	0	0	0	0
Pest	1	0	-1	-1	0	1
Specialist enemy	0	0	-1	0	0	0
Generalist enemy	0	0	0	-1	1	1
Edge density	0	0	0	0	-1	0
Non-crop habitat	0	0	0	0	0	-1

Table S3 Weighted feedback (*wF*) values calculated at system levels *1* to *6* for the 6-variable models of archetypes A1 and A2.

	wF_1	wF_2	wF_3	wF_4	wF_5	wF_6
**A1 model**	-1	-1	-1	-1	-1	-1
**A2 model**	-1	-1	-1	-1	-1	-1

Table S4 Classical adjoint of the negative $\mathbf{A°_1}$ matrix. Each table value corresponds to the sign of the equilibrium response of the row variable to an increase in a parameter of the growth rate function of the column variable.

	Crop yield	Pest	Specialist enemy	Generalist enemy	Edge density	Non-crop habitat
Crop yield	1	0	1	0	0	0
Pest	0	0	-1	0	0	0
Specialist enemy	1	1	1	-1	-1	-1
Generalist enemy	0	0	0	1	1	1
Edge density	0	0	0	0	1	0
Non-crop habitat	0	0	0	0	0	1

Table S5 Classical adjoint of the negative $\mathbf{A°_2}$ matrix. Each table value corresponds to the sign of the equilibrium response of the row variable to an increase in a parameter of the growth rate function of the column variable.

	Crop yield	Pest	Specialist enemy	Generalist enemy	Edge density	Non-crop habitat
Crop yield	0	-1	1	1	1	0
Pest	1	1	-1	-1	-1	0
Specialist enemy	0	0	1	0	0	0
Generalist enemy	0	0	0	1	1	1
Edge density	0	0	0	0	1	0
Non-crop habitat	0	0	0	0	0	1

S2 Stochastic differential equations

Archetype A1

The model is implemented as a set of stochastic differential equations (see below). However, model structure can most easily be explained by the following deterministic equations:

$$\frac{dP}{dt} = aP - \mu_P P - \frac{f_s PS}{P + K_s} - \frac{f_g PG}{P + K_g} + \upsilon_p (P_0 - P)$$

$$\frac{dS}{dt} = -\mu_S S + \frac{\varepsilon_s f_s PS}{P + K_s} + \upsilon_s (S_0 - S)$$

$$\frac{dG}{dt} = -\mu_G G + \upsilon_g (G_0 - G)$$

where P is the number of pests, S the number of specialist natural enemies (e.g., parasitoids) and G the number of generalist natural enemies in the crop, which suffer from natural mortality at rates μ_P, μ_S and μ_G, or have mean lifetime rates of $1/\mu_P$, $1/\mu_S$ and $1/\mu_G$, respectively. The pests suffer predation at rate

$$\frac{f_s PS}{P + K_s}$$

by the specialist natural enemies, who convert this resource with efficiency ε_s. The variables f_s and K_s parameterise the functional type II response, which describes the ability of the specialist enemies to detect and consume the pest. For the parameters shown, the average time taken to handle a prey item is $1/f_s$ and the rate at which the specialist enemy encounters prey per unit density is f_s/K_s.

Similarly, generalist natural enemies prey on the pest at rate

$$\frac{f_g PG}{P + K_g}$$

where G represents the generalist predator population size, and now f_g and K_g describe the generalists' ability to detect and consume the pest. Note in this model, the population size G of generalist predators within the crop is not increased by predation on pests, but is assumed to be controlled only by external factors independent of the pest population, e.g., the quality and extent of non-crop habitat and field edge density.

The influence of the wider environment beyond the crop, is described in terms of both inflow and outflow of organisms, e.g., for pests $\upsilon_p P_0$ and $\upsilon_p P$, respectively. In the deterministic model this results in net rates of inflow of pests $\upsilon_p(P_0 - P)$ and specialist $\upsilon_s(S_0 - S)$ and generalist natural enemies $\upsilon_g(G_0 - G)$. The terms P_0, S_0 and G_0 represent the ability of the non-crop habitat to provide pests and specialist and generalist natural enemies to the crop habitat, and thus are likely to be influenced by/reflect the quality and extent of non-crop habitat. The parameters υ_p, υ_s and υ_g describe connectivity between crop and non-crop habitat, potentially experienced differently by pests and specialist and generalist natural enemies.

Changes in landscape characteristics affect archetype A1 as follows (see Table S6). Increasing (decreasing) non-crop habitat increases (decreases) generalists via the source term G_0. Increasing (decreasing) edge density increases (decreases) dispersal of generalists υ_g and the source term G_0. The latter effect reflects the fact the increases in edge density reflect an increase in habitat, e.g., hedgerows, bunds etc. Specialists and pests are unaffected by the landscape characteristics.

Archetype A2

The model is implemented as a set of stochastic differential equations (see below). However, model structure can most easily be explained by the following deterministic equations:

$$\frac{dP}{dt} = aP - \mu_P P - \frac{f_s PS}{P + K_s} - \frac{f_g PG}{P + K_g} + \upsilon_p(P_0 - P)$$

$$\frac{dS}{dt} = -\mu_S S + \upsilon_s(S_0 - S)$$

$$\frac{dG}{dt} = -\mu_G G + \upsilon_g(G_0 - G)$$

The model for archetype A2 is the same as for archetype A1, except that now specialist natural enemy reproduction is not affected by predation of the pest. This is equivalent to assuming that $\varepsilon_s = 0$. The specialist natural enemy population is therefore controlled by the balance between migration in and out of the crop and mortality within the crop. In this respect, in archetype A2 the specialist population behaves in a similar way to the generalist population.

Changes in landscape characteristics affect archetype A2 as follows (see Table S6). Generalist enemies are affected by the landscape as under archetype A1, and specialists remain unaffected by landscape characteristics. In addition, increasing (decreasing) non-crop habitat increases (decreases) pests via the source term P_0, i.e., non-crop habitat increases inflow of pests to the crop. This is due to the transient nature of the pest under archetype A2, i.e. that it 'overwinters' outside the crop.

Parameter values for the models of archetypes A1 and A2 are given in Table S7.

Stochastic differential equation formulation of the model for archetypes A1 and A2

$$dP(t) = \left(aP - \mu_P P - \frac{f_s PS}{P + K_s} - \frac{f_g PG}{P + K_g} + \upsilon_p(P_0 - P) \right) dt$$

$$+ \sqrt{aP}\, dB_1(t) - \sqrt{\mu_P P}\, dB_2(t) - \sqrt{\frac{f_s PS}{P + K_s}}\, dB_3(t) - \sqrt{\frac{f_g PG}{P + K_g}\ dB_4(t)} + \sqrt{\upsilon_p P_0}\, dB_5(t)$$

$$- \sqrt{\upsilon_p P}\, dB_6(t)$$

$$dS(t) = \left(-\mu_S S + \frac{\varepsilon_s f_s PS}{P + K_s} + \upsilon_s(S_0 - S)\right) dt + \; -\sqrt{\mu_S S}\, dB_7(t) + \sqrt{\frac{\varepsilon_s f_s PS}{P + K_s}}\, dB_3(t) + \sqrt{\upsilon_s S_0}\, dB_8(t) - \sqrt{\upsilon_s S}\, dB_9(t)$$

$$dG(t) = \left(-\mu_g G + \upsilon_g(G_0 - G)\right) dt + \; -\sqrt{\mu_g G}\, dB_{10}(t) + \sqrt{\upsilon_g G_0}\, dB_{11}(t) - \sqrt{\upsilon_s S}\, dB_{12}(t)$$

Here, $dB_i(t)$ represent Gaussian white noise and for each t and $i = 1, \ldots 12$ are independent draws from $N\left(0, \sqrt{dt}\right)$.

Table S6 Representation of impacts of increasing landscape proportion of non-crop habitat (NCH) and field edge density (ED) on the models of archetypes A1 and A2.

**A1 model**	Pest	Specialists	Generalists	Model results
NCH	–	–	+ve G_0	See Figure S1
ED	–	–	+ve υ_g, G_0	See Figure S2
**A2 model**	Pest	Specialists	Generalists	Model results
NCH	+ve P_0	–	+ve G_0	See Figure S3
ED	–	–	+ve υ_g, G_0	See Figure S4

Table S7 Model parameters. The values shown are used for both archetype models, except where different values are indicated for archetypes A1 and A2.

**Parameters and default values**	**Pest**	**Specialists**	**Generalists**
Growth rate (day^{-1})	a = 0.15		
Death rates (day^{-1})	μ_P = 0.025	$\mu_S = \mu_P$	μ_G = $0.1\mu_S$
Migration rates (day^{-1})	$\upsilon_p = 0.1$	$\upsilon_s = 0.1$	$\upsilon_g = 0.1$
Source terms	$P_0 = 1.0$	$S_0 = 1.0$	$G_0 = 1.0$
		Predation Type II functional response	
Half saturation constant		$K_s = 60$	$K_g = 30$
Rate		$f_s = 50$	$f_g = 25$
Efficiency		ε_s = 0.44 (A1)<br>ε_s = 0.00 (A2)	0

Results

Here, the models described above are used to explore the impact of changing landscape proportion of non-crop habitat and field edge density for each archetype. Figures S1 and S3 show that the response of archetype A2 to increases in non-crop habitat is distinct from archetype A1. This reflects differences in both how non-crop habitat affects pests (see Table S6) and the numerical response of specialists to pest predation in the two archetypes. Contrasting Figures S2 and S4 show a different response when varying edge density, even though in this case the direct effect (see Table S6) is the same. This discrepancy therefore reflects differences in the numerical response of specialists between archetypes A1 and A2.

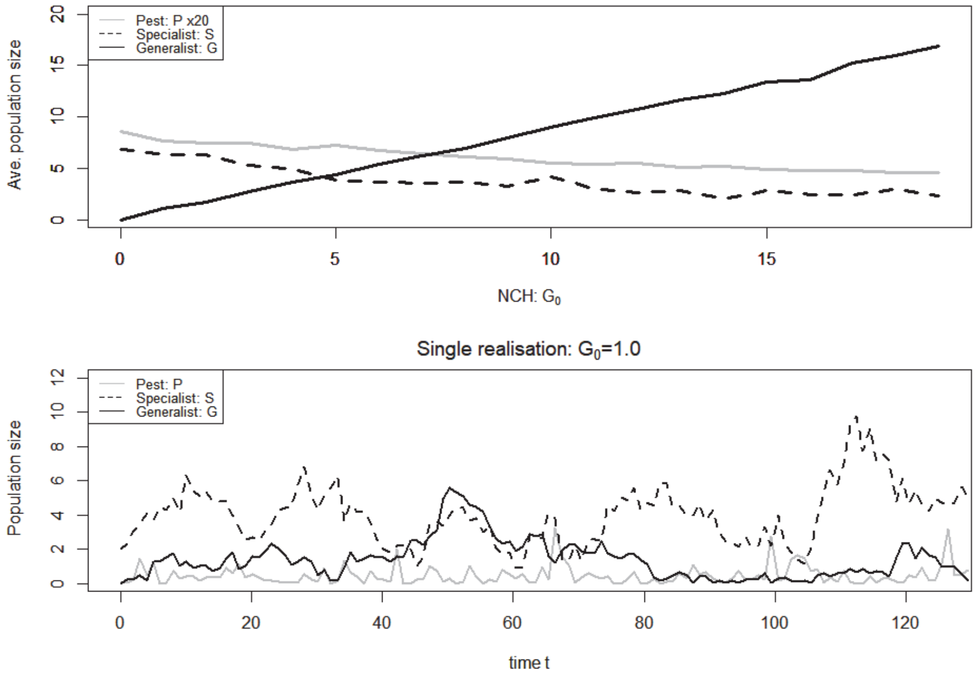

Figure S1 Model output for archetype A1 in response to varying non-crop habitat (NCH): $G_0 \in [0,20]$, with all other parameters fixed. Pest and specialists slightly decline with increasing NCH, whilst generalists increase.

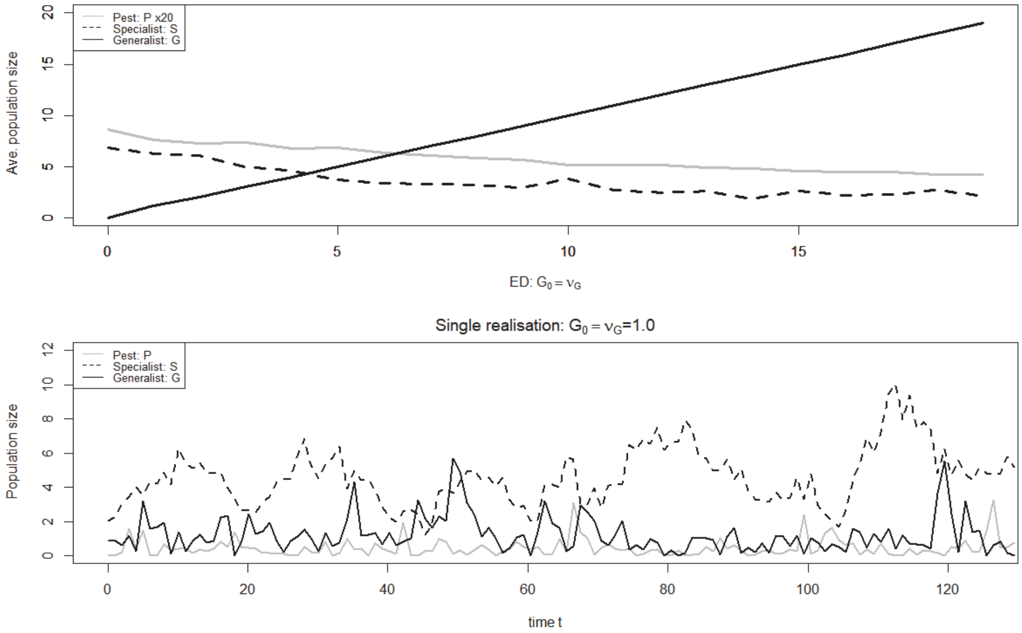

Figure S2 Model output for archetype A1 in response to varying edge density (ED): $G_0 = \upsilon_g \in [0,20]$, with all other parameters fixed. As ED increases, pests and specialist natural enemies decline.

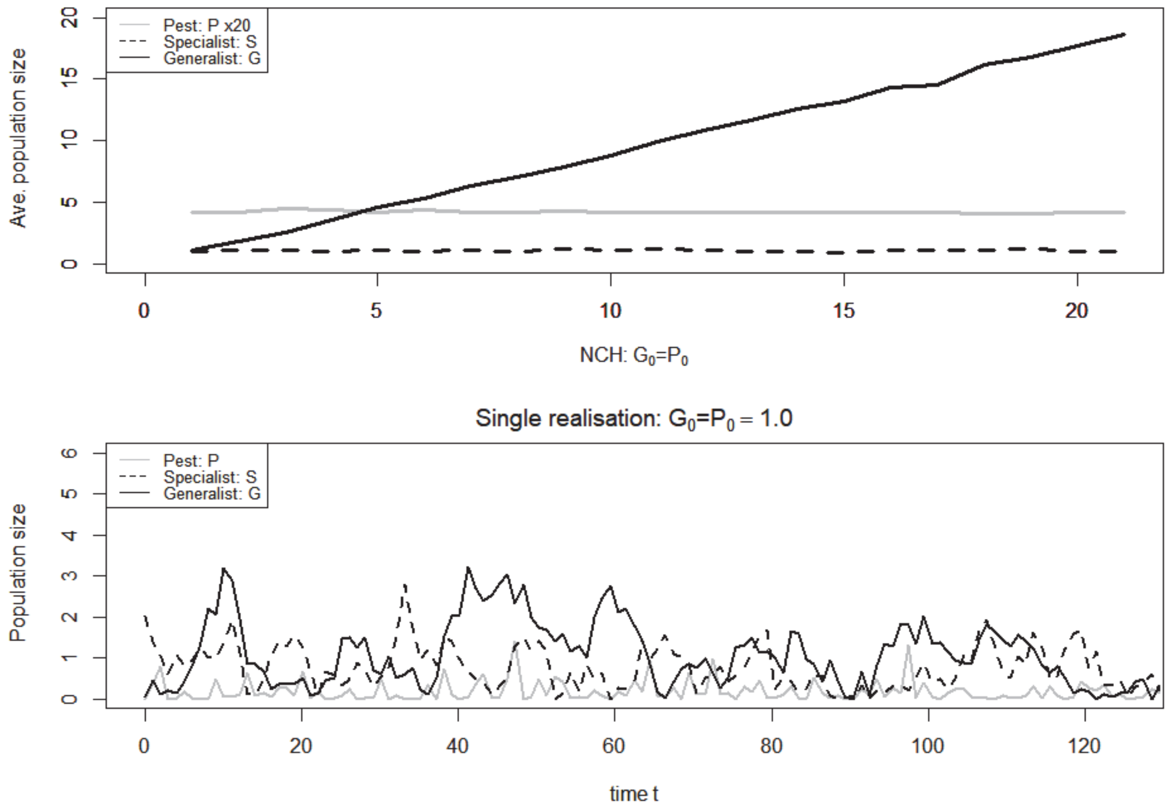

Figure S3 Model output for archetype A2 in response to varying non-crop habitat (NCH): $G_0 = P_0 \in [0,20]$, with all other parameters fixed, and the same migration rates for pests and specialist and generalist natural enemies $\upsilon_G = \upsilon_S = \upsilon_p = 0.1$. Generalist natural enemies increase with NCH, but specialists remain at low levels as NCH increases, and pest levels are relatively unaffected by NCH. This contrasts with archetype A1 (see Figure S1), where increasing NCH reduces pest levels.

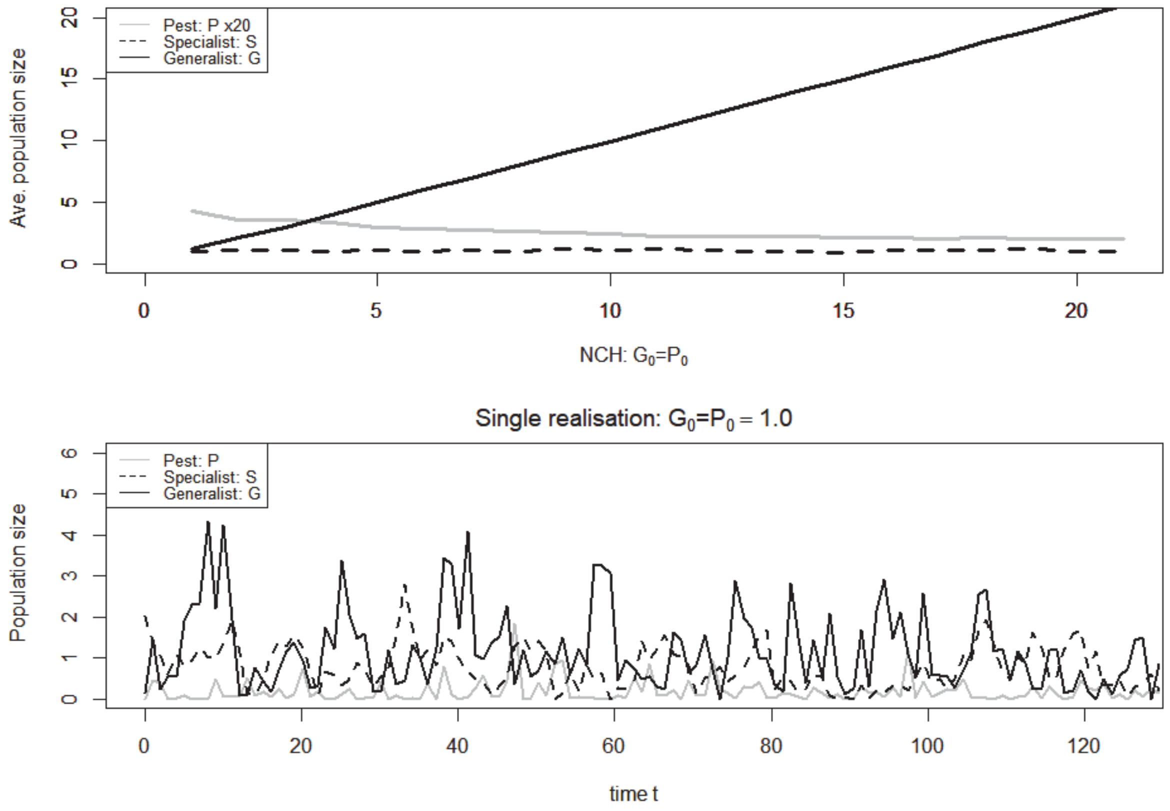

Figure S4 Model output for archetype A2 in response to varying edge density (ED): $G_0 = \upsilon_g \in [0,20]$, with all other parameters fixed. In comparison with Figure S2, we see a similar effect on pest, but no impact on specialist natural enemies as seen for archetype A1.

References

Dambacher, J.M., Li, H.W., Rossignol, P.A., 2002. Relevance of community structure in assessing indeterminacy of ecological predictions. Ecology 83, 1372–1385.

Dambacher, J.M., Luh, H.K., Li, H.W., Rossignol, P.A., 2003. Qualitative stability and ambiguity in model ecosystems. Am. Nat. 161, 876–888.

Maplesoft, 2017. Maple. Waterloo Maple Inc., Waterloo, Canada.

Novak, M., Yeakel, J.D., Noble, A.E., Doak, D.F., Emmerson, M., Estes, J.A., Jacob, U., Tinker, M.T., Wootton, J.T., 2016. Characterizing species interactions to understand press perturbations: what is the community matrix? Annu. Rev. Ecol. Evol. Syst. 47, 409–432.

Puccia, C.J., Levins, R., 1985. Qualitative modeling of complex systems: an introduction to loop analysis and time averaging. Harvard University Press, Cambridge, Massachusetts.

Westfahl, P., Heath, Z., Woodrow, C., 2002. PowerPlay Digraph Editor. Loop Group Dev Team, Corvallis, USA.
